# Single-cell transcriptomic analysis of flowering regulation and vernalization in Chinese cabbage shoot apex

**DOI:** 10.1093/hr/uhae214

**Published:** 2024-07-30

**Authors:** Yun Dai, Shifan Zhang, Jiantao Guan, Shaoxing Wang, Hui Zhang, Guoliang Li, Rifei Sun, Fei Li, Shujiang Zhang

## Abstract

In Chinese cabbage development the interplay between shoot apex activity and vernalization is pivotal for flowering timing. The intricate relationship between various cell types in the shoot apex meristem and their roles in regulating flowering gene expression in Chinese cabbage is not yet fully understood. A thorough analysis of single-cell types in the Chinese cabbage shoot apex and their influence on flowering genes and vernalization is essential for deeper insight. Our study first established a single-cell transcriptomic atlas of Chinese cabbage after 25 days of non-vernalization. Analyzing 19 602 single cells, we differentiated them into 15 distinct cell clusters using established marker genes. We found that key genes in shoot apex development and flowering were primarily present in shoot meristematic cells (SMCs), companion cells (CCs), and mesophyll cells (MCs). *MADS-box protein FLOWERING LOCUS C 2* (*BrFLC2*), a gene suppressing flowering, was observed in CCs, mirroring patterns found in *Arabidopsis*. By mapping developmental trajectories of SMCs, CCs, and MCs, we elucidated the evolutionary pathways of crucial genes in shoot apex development and flowering. The creation of a single-cell transcriptional atlas of the Chinese cabbage shoot apex under vernalization revealed distinct alterations in the expression of known flowering genes, such as *VERNALIZATION INSENSITIVE 3* (*VIN3*), *VERNALIZATION 1* (*VRN1*), *VERNALIZATION 2* (*VRN2*), *BrFLC*, and *FLOWERING LOCUS T* (*FT*), which varied by cell type. Our study underscores the transformative impact of single-cell RNA sequencing (scRNA-seq) for unraveling the complex differentiation and vernalization processes in the Chinese cabbage shoot apex. These insights are pivotal for enhancing breeding strategies and cultivation management of this vital vegetable.

## Introduction

Chinese cabbage (*Brassica rapa* L. ssp. *pekinensis*), also known as heading or wrapping cabbage, is a leafy vegetable from the crucifer family with significant economic value as a globally cultivated crop [1]. This vegetable is known for its rapid growth and development, making it crucial in agriculture. Its flowering timing is closely linked to environmental factors, especially the interaction between shoot apex activity and vernalization. Vernalization, the exposure to prolonged cold temperatures to trigger spring flowering, is vital for many temperate plants [2]. This process is key for optimal crop production and highlights the complexity of flowering time regulation mediated by vernalization. Understanding this mechanism in Chinese cabbage involves dissecting the cellular and molecular dynamics influenced by vernalization.

In the realm of plant developmental biology, the shoot apical meristem (SAM) of Chinese cabbage is a dynamic and complex structure that orchestrates the vegetative growth phase and transition to flowering. The molecular mechanisms of vernalization have been well studied in model plants, including *Arabidopsis*, identifying key regulators such as *FLC* (*FLOWERING LOCUS C*) and *VRN1* (*VERNALIZATION 1*) [3, 4]. However, the intricate processes and regulatory networks in Chinese cabbage are not yet fully understood. Single-cell RNA sequencing (scRNA-seq) provides a unique opportunity to dissect the vernalization-mediated mechanism of flowering time control. By providing a high-resolution view of the cellular complexity and regulatory networks, scRNA-seq enables a distinctive opportunity to evaluate the response to vernalization.

Although substantial research has explored the SAM regulatory mechanisms in model plants, including *Arabidopsis*, the processes in Chinese cabbage are less understood. Our study addresses this gap by employing innovative scRNA-seq techniques. Previous research has shown that vernalization involves the complex interplay of genetic and epigenetic modifications, leading to the stable repression of floral repressors and the activation of flowering pathways [2, 5]. However, these findings need to be expanded and validated in species such as Chinese cabbage to understand their universal applicability and species-specific variations.

The emergence of scRNA-seq technology has transformed our grasp of cellular complexity and regulatory mechanisms in plants, making it an ideal tool to explore vernalization-mediated flowering time control. Single-cell studies in plants are no longer limited to the leaves [6], shoot apex [7], stomata [8], and roots [9–11] of *Arabidopsis* but have gradually been extended to other species. scRNA-seq has been employed to uncover the cellular map of *Populus* xylem roots [12], to identify strong cell-type markers and specific regulatory programs in legume root cells [13], and to characterize key transcription factors (TFs) in allotetraploid peanut leaves [14]. The use of single cells has provided unique insights into organ growth and development, differentiation processes, tissue-specific responses to abiotic stress, cell-type-specific inheritance patterns, responses to biotic stress, distinct cell-type reactions to genetic changes, and the dynamics of cell cycle regulation. The ability of scRNA-seq to reveal cell-type-specific responses and regulatory programs makes it particularly suited for investigating the complex developmental processes influenced by vernalization in Chinese cabbage. This novel application of scRNA-seq is starting to shed light on these processes, suggesting that vernalization affects flowering time by modulating the activity and interaction of specific cell types within the shoot apex.

In this study, we aimed to bridge this knowledge gap by employing scRNA-seq to dissect the cellular landscape of the Chinese cabbage shoot apex. Our objective was to create a comprehensive single-cell transcriptomic atlas of Chinese cabbage that has undergone non-vernalization and to clarify the roles of distinct cell types in the regulation of flowering genes. By comparing our findings with available data from model species, such as *Arabidopsis*, we seek to uncover conserved and unique aspects of shoot apex development and flowering gene regulation. Additionally, we have constructed a single-cell map of Chinese cabbage during vernalization, highlighting the transformative potential of scRNA-seq in understanding the complex interplay of cellular responses during vernalization. This study not only aims to advance our understanding of the developmental biology of Chinese cabbage, but also seeks to provide valuable insights for the effective management and breeding of this important crop.

## Results

### Single-cell RNA sequencing of cells from Chinese cabbage shoot apex

**Figure 1:**
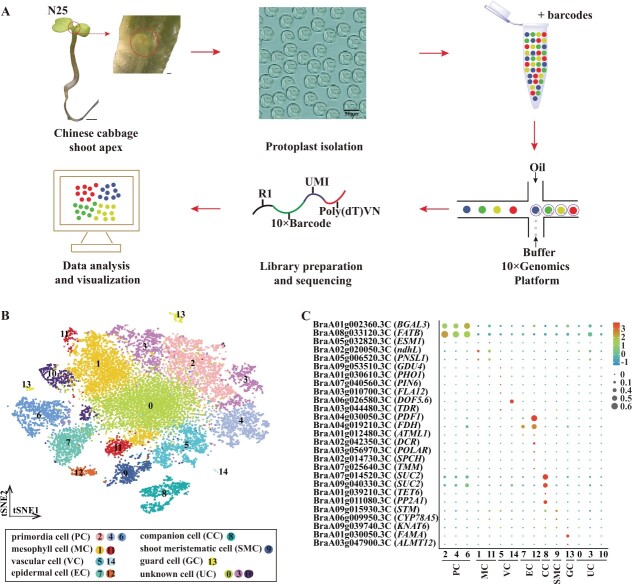
Generation of a cell atlas for the Chinese cabbage shoot apex. **A** This schematic illustrates the isolation of protoplast cells from the Chinese cabbage shoot apex and their subsequent placement on the 10× Genomics platform. Short scale bar represents 200 μm; long scale bar represents 1000 μm. **B**  *t*-SNE visualization shows 15 identified cell clusters in the Chinese cabbage shoot apex. Each dot represents an individual cell, with colors indicating the corresponding clusters. **C** Bubble plot demonstrating expression patterns and distributions of cluster-specific genes, aiding in cell type identification within the Chinese cabbage shoot apex. These plots show both the average expression level (by color) and the proportion of cells expressing each gene (by dot size).

To perform scRNA-seq on the Chinese cabbage shoot apex, we collected ~200 shoot apexes from 25-day-old non-vernalized (N25) Chinese cabbage and observed them under a stereomicroscope (M125, Leica, Germany). The cells were converted into protoplasts (Chinese cabbage shoot apex cells without cell walls) and analyzed using scRNA-seq on the 10× Chromium platform (Fig. 1A). A total of 19 602 individual cells were obtained after the cell filtering process (Supplementary Data Fig. S1, Supplementary Data Table S1), and 48 407 genes were identified (Supplementary Data Table S2). Different cell populations were identified using known marker genes and classified into 15 distinct clusters (Fig. 1B, Supplementary Data Tables S3 and S4).

The *t*-SNE (*t*-distributed stochastic neighbor embedding) and UMAP (uniform manifold approximation and projection) algorithms were used to illustrate local similarities and overall cell population structures (Fig. 1B, Supplementary Data Fig. 1B). We used reported marker genes to identify the cell types of different clusters of Chinese cabbage shoot apexes and revealed seven broad populations: primordial cells (PCs), mesophyll cells (MCs), vascular cells (VCs), epidermal cells (ECs), companion cells (CCs), shoot meristematic cells (SMCs), and guard cells (GCs) (Fig. 1B and 1C). The PC population consisted of three clusters (clusters 2, 4, and 6) in which *BETA-GALACTOSIDASE 3* (*BGAL3*) and *FATTY ACYL-ACP THIOESTERASES B* (*FATB*) were predominantly expressed [15]. The specific genes *GDSL esterase/lipase* (*ESM1)*, *NAD(P)H-quinone oxidoreductase subunit L* (*ndhL*), and *PHOTOSYNTHETIC NDH SUBCOMPLEX L1* (*PNSL1*) [7, 16] were detected in the MC population (clusters 1 and 11). VC was assigned to clusters 5 and 14, in which the following genes were expressed: *GLUTAMINE DUMPER 4* (*GDU4*), *PHOSPHATE TRANSPORTER 1* (*PHO1*), *PIN-FORMED 6* (*PIN6*), *FASCICLIN-LIKE ARABINOGALACTAN-PROTEIN 12* (*FLA12*), *DNA BINDING WITH ONE FINGER 5*.*6* (*DOF5*.*6*), and *TDIF RECEPTOR* (*TDR*) [17–22]. We annotated two clusters (7 and 12) as the EC population because the following genes were overrepresented: *PROTODERMAL FACTOR 1* (*PDF1*), *FIDDLEHEAD* (*FDH*), *MERISTEM LAYER 1* (*ATML1*), *DEFECTIVE IN CUTICULAR RIDGES* (*DCR*), *POLAR*, *SPEECHLESS* (*SPCH*), and *TOO MANY MOUTHS* (*TMM*) [23–28]. Clusters 8 and 13 comprised CC and GC populations, respectively. The CC and GC marker genes included *SUCROSE-PROTON SYMPORTER 2* (*SUC*2), *TETRASPANIN 6* (*TET6*), *PHLOEM PROTEIN2-A1* (*PP2A1*), *FAMA*, and *ALUMINUM-ACTIVATED MALATE TRANSPORTER 12* (*ALMT12*) [29–33]. The SMC population included cluster 9 and transcripts for *SHOOT MERISTEMLESS* (*STM*), which is essential for establishing and maintaining SAM in *Arabidopsis* [34]. *CYTOCHROME P450* (*CYP78A5)* and *KNOTTED1-LIKE HOMEOBOX GENE 6 (KNAT6*) [35] were similarly highly expressed in cluster 9, thereby identifying cluster 9 as a SAM-associated cell population. Two marker genes, *PNSL1* (BraA05g006520.3C) and *SUC2* (BraA07g014520.3C), were chosen for RNA *in situ* hybridization, and strong hybridization signals were observed in the shoot apex structure (Supplementary Data Fig. S2). Using these marker genes to identify these cell clusters provided new insights into the cell types in the shoot apex of Chinese cabbage and provided direction for subsequent studies of flowering-related genes in the shoot apex.

### Characterization of the novel marker gene in each cell cluster

**Figure 2:**
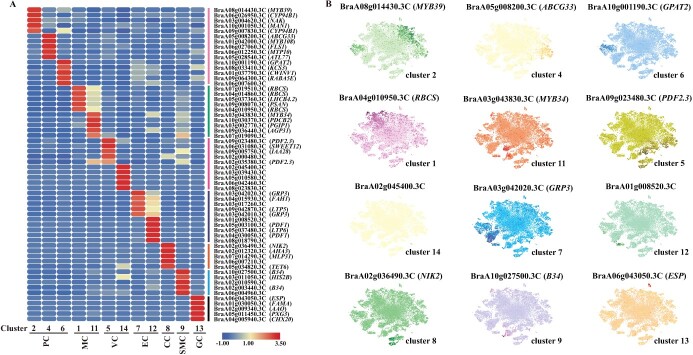
Discovery of novel marker genes in cell-type clusters. **A** The heat map displays the top five DEGs with the highest log^2^ TPM expression levels in each subcluster (Supplementary Data Table S2). Red signifies high expression levels, while blue denotes low expression levels. **B** Expression patterns of 12 new marker genes distributed on the *t*-SNE map. The color gradient in each *t*-SNE plot represents the expression level of the gene, with darker points indicating higher expression and lighter points indicating lower expression.

To identify marker genes, we compared the associated upregulated differentially expressed genes (DEGs) across one cluster with those in other clusters, and the DEGs were distributed in a range from 156 to 1797 per cluster (Supplementary Data Table S5). We analyzed the annotated clusters using Gene Ontology (GO) and found that the PC population with the largest share of DEGs was mainly annotated in ‘response to stimulus’, ‘response to biotic stimulus’, and ‘response to stress’. Consistent with the cell cluster classification results, GO term analysis of the MC population was mostly organelle-related (Supplementary Data Fig. S3). Only a few plants, including *Arabidopsis*, rice, and peanut, have databases for cell type identification. The marker genes from these databases were not entirely sufficient for cell type identification in Chinese cabbage, thus making it a critical step in Chinese cabbage to determine cell type-specific expression of new genes. After analysis of the DEGs, we identified and designated the five most highly expressed genes in each cluster as new marker genes for these clusters (Supplementary Data Table S5, Supplementary Data Fig. S4). In total, we identified 60 new marker genes, which were visualized in a heat map (Fig. 2A). The top marker gene for each cluster was determined using *t*-SNE mapping (Fig. 2B). These new marker genes will be helpful in the identification of different cell types in future shoot apex studies in Chinese cabbage and even cruciferous plants.

### Cellular dissection of shoot apex gene regulation in Chinese cabbage and *Arabidopsis*

**Figure 3:**
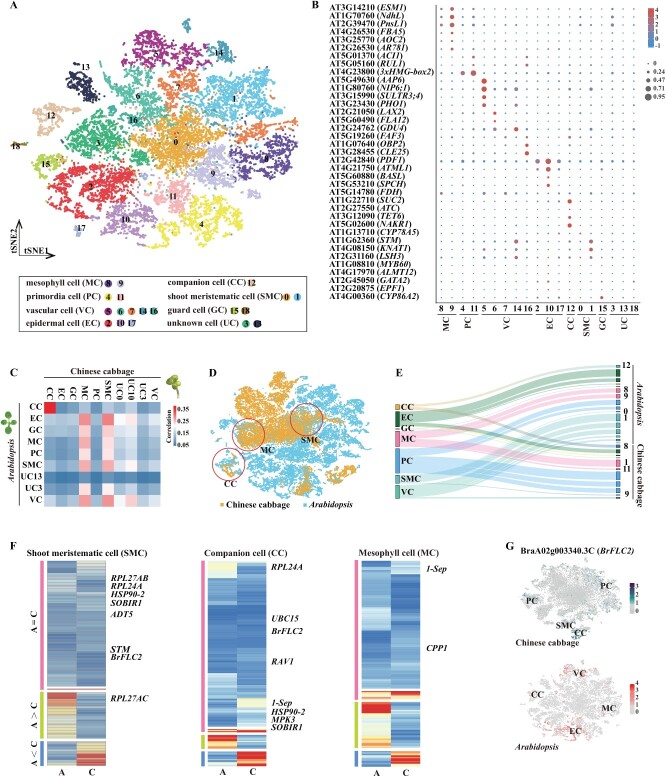
Comparison of Chinese cabbage and *Arabidopsis* shoot apexes at single-cell resolution. **A**  *t*-SNE visualization shows 19 identified cell clusters in the *Arabidopsis* shoot apex. Each dot represents an individual cell, with colors indicating the corresponding clusters. **B** Bubble plot demonstrating expression patterns and distributions of cluster-specific genes, aiding in cell type identification within the *Arabidopsis* shoot apex. These plots show both the average expression level (by color) and the proportion of cells expressing each gene (by dot size). **C** Pairwise correlations of Chinese cabbage (top) and *Arabidopsis* (left) shoot apex cell clusters, with dots indicating statistically significant correlations. CC, companion cell; EC, epidermal cell; GC, guard cell; MC, mesophyll cell; PC, primordial cell; SMC, shoot meristematic cell; VC, vascular cell; UC, unknown cell. **D**  *t*-SNE plot depicting cell clusters in Chinese cabbage and *Arabidopsis* shoot apex cells, with dotted circles marking common MC, CC, and SMC clusters. **E** Sankey diagrams showing the similarity of Chinese cabbage to *Arabidopsis* across cell clusters. All clusters were generated after merging the *Arabidopsis* and Chinese cabbage scRNA data on the left (Supplementary Data Fig. S5B). Cluster numbers for Chinese cabbage (Fig. 1B) and *Arabidopsis* (Fig. 3A) shoot apex cells are given on the right. **F** Gene clustering of the SMC, CC, and MC clusters. A = C indicates genes with conserved expression in *Arabidopsis* (A) and Chinese cabbage (C), and A > C and A < C indicate genes specifically expressed in *Arabidopsis* or Chinese cabbage, respectively. The displayed genes are related to shoot development and flowering genes. Red shows high expression levels, while blue represents low expression levels. **G**  *BrFLC2* and *AtFLC *expression pattern in *Arabidopsis* and Chinese cabbage, as plotted using *t*-SNE.

*Arabidopsis* shoot apex scRNA-seq datasets were already available [7]. We investigated the conservation and differentiation of Chinese cabbage and *Arabidopsis* shoot apex cell types in various genera within the same family. We also explored the distribution of conserved and specifically expressed genes in conserved cell types. We used the published *Arabidopsis* shoot apex shoot4 scRNA-seq data (BioProject PRJCA003094) and reperformed cell clustering and cell group identification on this dataset using the same analysis pipeline for Chinese cabbage and identified 19 clusters (Fig. 3A and 3B, Supplementary Data Table S6). We then identified one-to-one homologous genes between Chinese cabbage and *Arabidopsis* using OrthoMCL [36] to compare gene expression at the cellular level (*n* = 19 145, Supplementary Data Table S7). A pairwise comparison of the Chinese cabbage and *Arabidopsis* cell populations revealed significant correlations between the clusters corresponding to CC. The correlation of MC, SMC, and VC clusters was also found in the corresponding clusters of Chinese cabbage and *Arabidopsis* (Fig. 3C). Interestingly, the MC and SMC clusters of Chinese cabbage also correlated with other cell clusters in *Arabidopsis* (Fig. 3C). The results offer valuable insights into evolutionary conservation and cell divergence in Chinese cabbage and *Arabidopsis*.

Next, we integrated the shoot apex scRNA-seq data of Chinese cabbage and *Arabidopsis* for cell clustering analysis, which yielded 44 430 cells and 19 145 genes and revealed 20 cell clusters (Supplementary Data Fig. S5, Supplementary Data Table S8). As Chinese cabbage and *Arabidopsis* shoot apexes were correlated with CC, MC, and SMC cell populations (Fig. 3D and 3E), we explored genes associated with shoot development and flowering in these three cell populations. For the three cell populations, SMC, CC, and MC (Fig. 3F, Supplementary Data Tables S9, S10, and S11), gene clustering analyses identified a set of conserved expression patterns and specific expression genes in Chinese cabbage and *Arabidopsis*. We found that genes related to shoot development and flowering were primarily present in conserved expression regions (A = C) in Chinese cabbage and *Arabidopsis* (Fig. 3F). Many previous studies in maize (*Zea mays*), *Arabidopsis*, and other species have revealed the importance of the KNOXI transcription factor SHOOT MERISTEMLESS (STM) in the establishment and maintenance of the SAM [37–40]. Here, the *STM* genes of Chinese cabbage and *Arabidopsis* belonged to the conserved expression genes of the SMC cell population, and it was evident that the *STM* gene also played a significant role in the SMCs of Chinese cabbage (Fig. 1C). In *Arabidopsis*, *MADS-box protein FLOWERING LOCUS C* (*FLC*) acts by repressing a series of flowering genes to suppress rapid flowering [41]. Four homologous *FLC* genes (*BrFLC1*, *BrFLC2*, *BrFLC3*, and *BrFLC5*) were identified and validated in Chinese cabbage compared with *Arabidopsis* [42, 43]. It was evident that, in contrast to other *BrFLC* types, *BrFLC2* (BraA02g00340.3C) was the most similar class with respect to *AtFLC* and was present in multiple cell populations. In contrast, *BrFLC2* was not only present in the conserved cell population CC but also in the PC and SMC populations in Chinese cabbage and in the EC, MC, and VC populations in *Arabidopsis* (Fig. 3G). This suggests that *BrFLC2* is important in both Chinese cabbage and *Arabidopsis*. The inter-species comparison analysis revealed conserved expression of genes between the two species, suggesting that the shoot apexes of the different species have conserved characteristics.

**Figure 4:**
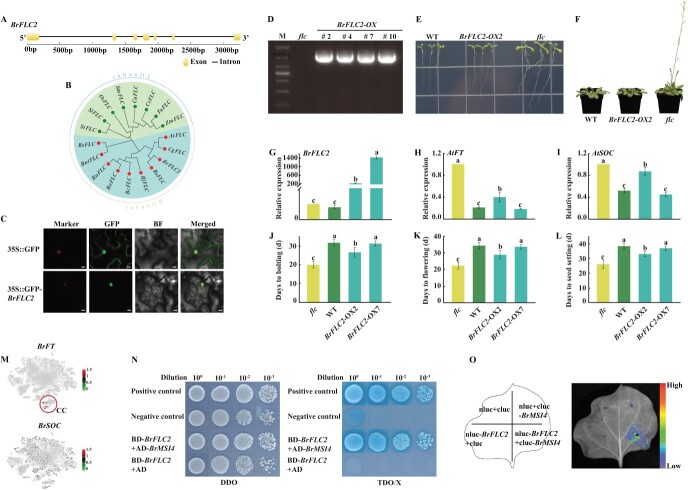
Preliminary analysis of the molecular function of Chinese cabbage *BrFLC2*. **A** Illustration of *BrFLC2* genome structure. **B** Phylogenetic tree of *BrFLC2* homologs in various plant species. **C** Subcellular localization of BrFLC2 protein in the tobacco nucleus. Scale bar, 25 μm. **D**  *BrFLC2* coding sequences from *BrFLC2*-OX lines cloned by PCR. **E**, **F** Phenotypes of *BrFLC2*-OX, *flc*, and WT lines grown in medium for 10 and 25 days after planting. **G**–**I** Relative expression of *BrFLC2*, *AtFT*, and *AtSOC* in *BrFLC2*-OX, *flc*, and WT lines. Error bars indicate the standard error (*n* = 3). **J**–**L** Days to bolting, flowering, and seed setting in *BrFLC2*-OX, *flc*, and WT lines. Error bars indicate the standard error (*n* = 10). **M** Expression pattern of *BrFT* and *BrSOC* in Chinese cabbage, as plotted on *t*-SNE. **N** Transcriptional activation function of BrFLC2 and BrMSI4 in yeast, with DDO representing SD/−Trp/−Leu, and TDO/X representing SD/−Trp/−His/−Leu medium supplemented with X-α-gal. **O** BrFLC2 and BrMSI4 interaction in tobacco epidermal cells, demonstrated by the luciferase complementation assay.

Here, we discovered that the *BrFLC2* (BraA02g00340.3C) gene is highly conserved in both *Arabidopsis* and Chinese cabbage and is present in numerous cell populations. We focused on understanding the mechanism and function of *BrFLC2*, a gene encoding a MADS-box protein involved in flowering. The coding sequence of Chinese cabbage *BrFLC2* was 591 bp and contained seven exons and six introns (Fig. 4A). Phylogenetic analysis showed that *BrFLC2* was similar to *RsFLC*, *AtFLC*, and *CgFLC* in group II (Fig. 4B). Experiments revealed that BrFLC2 proteins localize in the nucleus when expressed in tobacco leaves (Fig. 4C). We further explored the function of *BrFLC2* by overexpressing it in *Arabidopsis flc* mutants (Fig. 4D). This led to slower growth and delayed flowering compared with *flc* mutants, with the growth rate and flowering time being similar to those of the wild type (WT) (Fig. 4E and 4F). The expression of *BrFLC2* in the *BrFLC2*-OX (overexpression) line was significantly higher compared with the *flc* and WT lines, where *BrFLC2* expression was negligible, approximately zero. Additionally, the expression of the flowering genes *AtFT* and *AtSOC* in the *BrFLC2*-OX line was significantly lower than in the *flc* line, tending towards the levels observed in the WT. This indicates that the ectopic expression of *BrFLC2* affected the expression of *Arabidopsis*-related flowering genes (Fig. 4G–4I). Simultaneously, we discovered that the *BrFT* gene in Chinese cabbage was exclusively expressed in the CC population, while *BrSOC* was expressed in all cell groups (Fig. 4M). This suggests that *BrFLC2* influenced the expression of *BrFT* in the CC population of Chinese cabbage. At the same time, *BrFLC2* overexpression increased the numbers of days to bolting, flowering, and seed setting, similar to the WT (Fig. 4J–4L). Further experiments, including yeast two-hybrid (Y2H) and luciferase complementation (LUC) assays, identified an interaction between BrFLC2 and BrMSI4 (Fig. 4N and 4O). Initially, we conducted yeast two-hybrid (Y2H) screening and identified 23 proteins that interacted with BrFLC2 (Supplementary Data Table S12). Following a comprehensive literature search and functional annotation, we found that BrMSI4 was associated with flowering regulation. BrMSI4 acts as a DDB1- and CUL4-associated factor that interacts with the CLF-polycomb repressive complex 2 (PRC2) to repress FLC expression [44]. Consequently, we selected *BrMSI4* for further validation due to its potential role in flowering control. This study enhances our understanding of *BrFLC2*’s function in the flowering process of both Chinese cabbage and *Arabidopsis*, indicating that the CC cell group might be crucial in plant flowering and merits additional research.

### Analysis of pseudo-time trajectories in Chinese cabbage shoot apex cells

**Figure 5:**
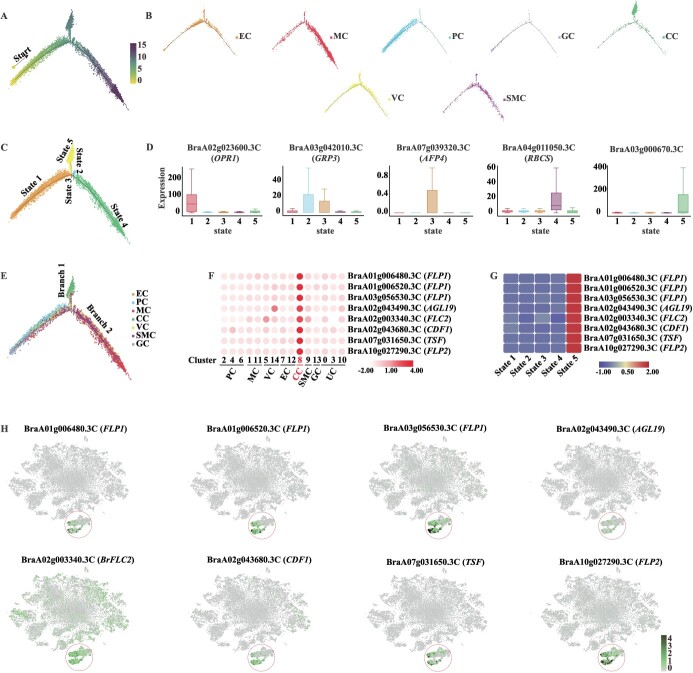
Pseudo-time trajectory analysis of cell types in the Chinese cabbage shoot apex. **A** Development trajectory of all shoot apex cells and the placement of each cell cluster in the trajectory map. **B** Placement of each individual cell cluster in the trajectory map. **C** Trajectory analysis dividing single cells into five differentiation states. **D** Pseudo-time trajectory analysis of five key marker genes’ expression patterns across five states. **E** Cell distribution within each cluster and pseudo-time trajectory. **F** Heat map showing the average expression levels of flowering genes across all cell trajectories. Red represents a high expression level. **G** Heat map showing average expression levels of flowering genes in five states, with red representing high expression and blue representing low expression. **H** Distribution of expression of eight flowering genes in the *t*-SNE map and heat map.

To examine the spatial and temporal distribution of individual shoot apex cells in Chinese cabbage, we used Monocle 2 software [45] for pseudo-time trajectory analysis. This approach allowed us to illustrate the placement of each cell cluster along the main stem (Fig. 5A and 5B, Supplementary Data Table S13). The pseudo-time analysis also identified five key marker genes (*OPR1*, *GRP3*, *AFP4*, *RBCS*, and BraA03g00670.3C) (Fig. 5D, Supplementary Data Table S14) that can categorize all individual cells into five unique states (Fig. 5C) of shoot apex development and differentiation.

In our pseudo-time trajectory analysis, two branches were found near the main stem: branch 1 (states 1, 3, 4, and 2 versus states 1 and 5) and branch 2 (states 1, 3, and 4 versus states 1, 3, and 2) (Fig. 5E). The differentiation of branches exhibited a type of cell heterogeneity over pseudo-time. In the two cell differentiation branches there were 4747 DEGs, 3974 in branch 1 and 773 in branch 2 (Supplementary Data Table S15). Through GO analysis, it was found that branch 1 mainly focused on ‘stimulus,’ ‘stress’, and ‘death’ with differences in GO enrichment of DEGs compared with branch 2 (Supplementary Data Fig. S6A and S6B). Based on the Kyoto Encyclopedia of Genes and Genomes (KEGG) presentation, the pathways enriched in branch 1 were primarily involved in ‘ribosome,’ ‘glutathione metabolism’, ‘oxidative phosphorylation’, and the ‘MAPK signaling pathway-plant’, while branch 2 was highly concentrated in the ribosome (Supplementary Data Fig. S6C and S6D). The branching provided a more intuitive observation for visualizing changes in cell expression. A total of 32 DEGs associated with the flowering and shoot development in the two differently branches were identified (Supplementary Data Fig. S6E). Interestingly, nearly all of these DEGs (31) were in branch 1, and 1 DEG (*1-sep*) was present in both branches (Supplementary Data Fig. S6E). The CC population was also enriched for several flowering and shoot development genes that were highly expressed in the CC population compared with other cell populations, with correspondingly high expression in state 5 (Fig. 5F–H). The main pseudo-time trajectory pathway for shoot development and flowering-related genes was shown to be the CC population differentiation pathway. This also corroborated our conclusion drawn from cross-species analysis that a large number of genes associated with flowering might have existed in the CC population.

### Differentiation trajectories of shoot and flowering genes

**Figure 6:**
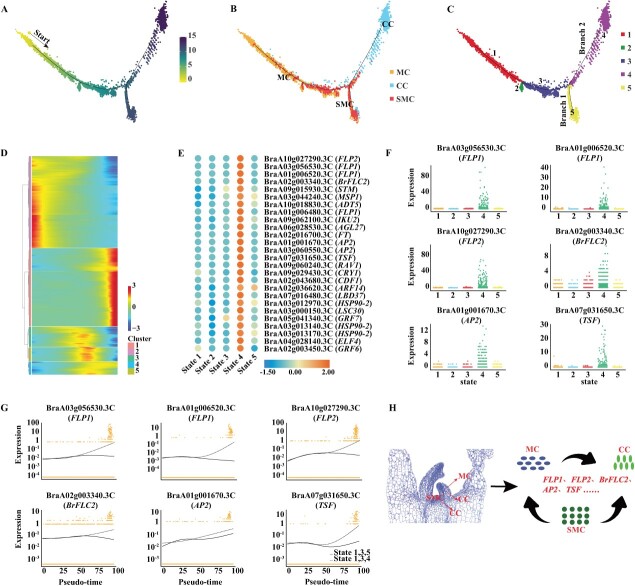
Developmental trajectory of companion cells from mesophyll cells and shoot meristematic cells. **A**–**C** Distribution of cell clusters, differentiation states, and branches along the pseudo-time trajectory of mesophyll development. **D** Clustering and expression dynamics of DEGs along the main stem of the pseudo-time trajectory. **E** Heat map showing average expression of relevant genes across five cell differentiation states, with red representing high expression and blue denoting low expression. **F** Expression distribution of six representative flowering genes in the cell differentiation state. **G** Expression distribution of six representative flowering genes in different branches. **H** A putative model for the developmental and differentiation patterns of companion cells from mesophyll cells and shoot meristematic cells.

Many flowering and shoot development genes existed in the MC, SMC, and CC populations, and we speculated that these three cell populations had interdependent developmental relationships. To test this hypothesis, the developmental trajectories of MC, SMC, and CC cell clusters were analyzed (Fig. 6A). By studying the differentiation orientation of the cells along the timeline, pseudo-time trajectory data revealed that SMCs and MCs accompany each other (Fig. 6B). However, SMCs are essentially undifferentiated cells within the plant growth point capable of differentiating into MCs, suggesting that MCs can arise from SMCs at any moment and location. Positioned at the initial segment of the timeline, MCs may also originate from other cell types, such as PCs. Simultaneously, CCs have the potential to differentiate from both SMCs and MCs (Fig. 6B). The backbone along the pseudo-time trajectory was divided into five states and two branches (Fig. 6C). Monocle 2 [45] is adept at harnessing the gene expression signals present across all cells, and by analyzing the gene expression profiles characteristic of cells in distinct differentiation states, it adeptly identifies DEGs across various states and branches of differentiation. As a result, the pseudo-time trajectory of the main stem revealed 3862 DEGs (Fig. 6D, Supplementary Data Table S16), and 2109 DEGs were identified in the two branches, with 485 DEGs in branch 1 and 1624 DEGs in branch 2 (Supplementary Data Table S17). Sixty genes related to flowering and shoot development were screened from the DEG profile, and most of the related genes were highly expressed in the CC population, i.e. state 4 (Fig. 6E). We then selected six representative flowering genes for description in state 4 (companion cell population) (Fig. 6F). The six representative genes were not only highly expressed in state 4, but with the pseudo-time trajectory their expression was also more skewed towards branch 2 (states 1, 3, and 4) (Fig. 6G). The SMC population, representative of SAM tissue cells in Chinese cabbage, was theoretically presumed to predominantly encompass genes related to flowering and shoot development. These flowering and shoot development genes were primarily observed in the CC population, exhibiting high expression levels. This observation led to the speculation that the hypothesized differentiation trajectory of the CC population might not exclusively originate from MCs but could also differentiate from SMCs, as depicted in Fig. 6H. Overall, this trajectory-based analysis delineates a hypothetical model for the differentiation of MC, SMC, and CC cell types, simultaneously reaffirming the importance of the CC population in regulating genes crucial for flowering and shoot development.

### Vernalization-induced transcriptomic changes vary among cell types

**Figure 7:**
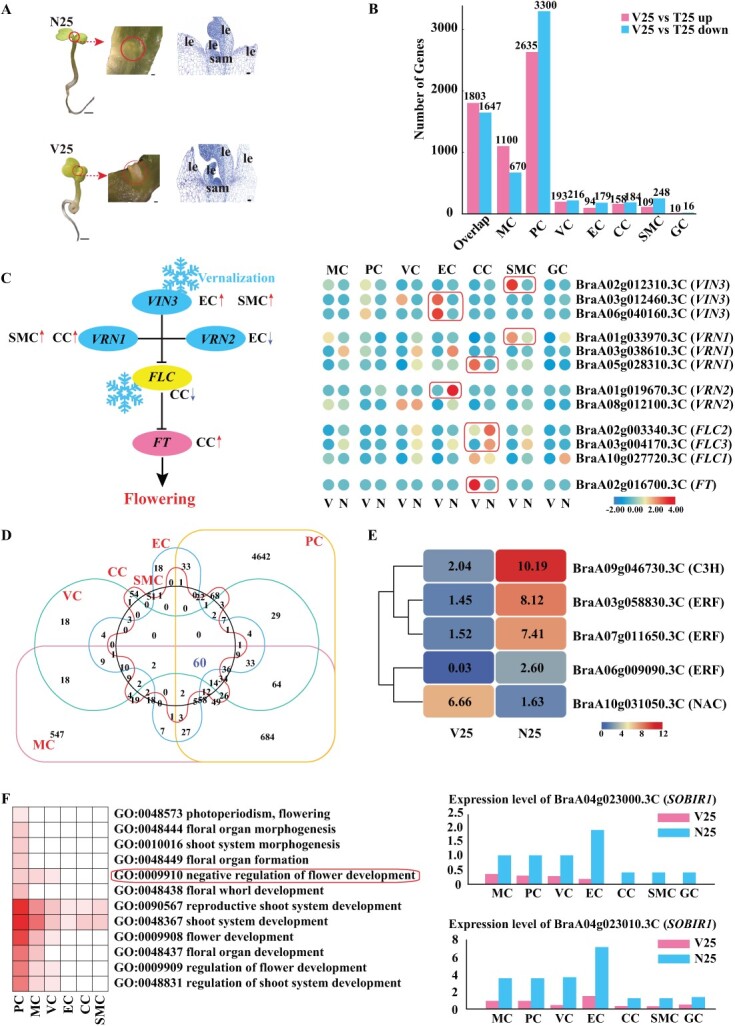
Differential gene expression patterns across various cell types during vernalization. **A** Chinese cabbage shoot apex sample pattern maps for control and vernalization treatments Short scale bar, 200 μm; long scale bar, 1000 μm; le, leaf or leaf primordium. **B** DEGs between control and vernalization treatments in different cell types. **C** Average expression and flowchart of each gene of the vernalization pathway in different cell types. V represents vernalization; NV represents non-vernalization. Red and blue represent high and low expression levels, respectively. **D** Venn diagram showing the distribution of overlapping DEGs between different cell types. **E** Relative expression of TFs in two samples in each cell type. Red signifies high expression levels, while blue indicates low expression levels. **F** GO-annotated DEG information related to flowering and shoot development and expression levels of *SOBIR1* genes in different cell types of the two samples. The depth of red represents the number of DEGs.

To investigate the cellular heterogeneity of the Chinese cabbage shoot apex in response to vernalization, we isolated protoplasts from the shoot apex vernalized for 25 days (V25), as detailed in Supplementary Data Table S18, with N25 serving as the control (Fig. 7A). Notably, a significant disparity in the number of cells captured between N25 and V25 prompted a cell frequency analysis and sample cluster recognition. This analysis confirmed regular cell frequency and essentially identical cell types in both samples (Supplementary Data Fig. S7). Furthermore, differential expression analysis of genes between the groups revealed no excessive deviation (Supplementary Data Fig. S7A). This finding aligns with previous studies [46, 47], which noted a substantial difference in the number of cells among single-cell groups, allowing our comprehensive analysis to proceed as expected. Utilizing subpopulation data from both samples, we conducted an intergroup differential analysis. DEGs were identified for each cell type under the control and vernalization treatments, considering a difference in the mean expression level of |log_2_FC| ≥ 0.36 and *P* < 0.05 (Fig. 7B, Supplementary Data Table S19). Excluding overlap and unknown cell clusters, compared with N25, the PC cluster of V25 exhibited the highest number of DEGs (2635 upregulated, 3300 downregulated), followed by the MC cluster (1100 upregulated, 670 downregulated), with the GC cluster displaying the fewest DEGs (10 upregulated, 16 downregulated).

Our focus was specifically on a cohort of genes intimately associated with vernalization, namely *VERNALIZATION INSENSITIVE 3* (*VIN3*), *VERNALIZATION 1* (*VRN1*), *VERNALIZATION 2* (*VRN2*), *BrFLC*, and *FLOWERING LOCUS T* (*FT*) [41, 48, 49], across various cell types in two samples. We identified three *VIN3* genes, two of which (BraA03g012460.3C and BraA06g040160.3C) exhibited high expression in the V25 samples of the EC population and one (BraA02g012310.3C) was present in the SMC population of the V25 samples (Fig. 7C). Additionally, BraA01g033970.3C (*VRN1*) and BraA05g028310.3C (*VRN1*) showed high expression in the SMC and CC populations of the V25 samples, respectively. In contrast, the expression of BraA05g038610.3C (*VRN1*) was marginally higher in the N25 samples of the MC, VC, and EC populations than in V25. Of the two *VRN2* genes, only BraA01g019670.3C was highly expressed in the EC population of the NV (non-vernalized) samples. Among the detected *FL*C genes, only *FLC2* and *FLC3* (BraA03g004170.3C) adhered to vernalization-regulated expression, which was substantially expressed in the CC population of the V25 sample. Conversely, the flowering gene *FT* (BraA02g016700.3C) was notably expressed in the CC of V25 due to vernalization, which aligns with the notion that vernalization fosters the expression of flowering genes.

We compared DEGs across six cell populations—MC, PC, VC, EC, CC, and SMC—and identified 60 DEGs in all populations between N25 and V25 (Fig. 7D, Supplementary Data Table S20). GO and KEGG analyses of these genes predominantly revealed significant enrichment in categories related to environmental adaptation and ‘stimulus’ responses (Supplementary Data Fig. S8A and S8B), signifying vernalization’s notable influence across various cell types. Among these shared differential genes, we identified five TFs: three ERFs, one C3H, and one NAC. The *NAC* gene was more highly expressed in the V25 samples, whereas the other four TFs showed higher expression in the N25 samples (Fig. 7E). Additionally, we conducted a detailed screening of DEGs in each cell type of Chinese cabbage for genes associated with shoot development and flowering, followed by GO annotation. Our findings revealed that genes linked to ‘reproductive shoot system development (GO:0090567)’ and ‘shoot system development (GO:0048367)’ were differentially expressed in all populations except GC. Moreover, two genes with a GO enrichment of ‘negative regulation of flower development (GO:0009910)’—BraA04g023000.3C (*SOBIR1*) and BraA04g023010.3C (*SOBIR1*)—exhibited significant differences in the PC, MC, and VC clusters of the two samples. Investigating their reduced expression in other cell populations due to vernalization, it appeared that *SOBIR1* may function similarly to *FLC* genes, inhibiting flowering under normal conditions and promoting flowering when expression is reduced due to vernalization.

## Discussion

This study provides a detailed single-cell atlas of the vegetative shoot apex in Chinese cabbage, revealing the complex cellular architecture of this non-model plant. Employing advanced scRNA-seq technology, we explored the cellular diversity within and across cell types in the plant. This innovative approach marks a significant stride in the study of complex biological processes, especially in non-model species. We meticulously characterized numerous cell clusters and state variations in the shoot apex, driven by a wide range of cell marker genes. This investigation not only demonstrates the feasibility of applying scRNA-seq to non-model organisms but also expands the potential for functional studies in the shoot apex of such species. Our findings underscore the pivotal role of vernalization in its developmental processes. The insights gained are crucial in enhancing our understanding of vernalization, opening up potential avenues for developing varieties with optimized flowering times and increased environmental adaptability.

We evaluated the pivotal role of the shoot apex in the growth and development of Chinese cabbage, a widely cultivated leafy vegetable. To this end, we utilized shoot apex data from the N25 treatment, analyzing 19 602 individual cells and 48 407 genes. These were classified into 15 distinct cell clusters based on specific marker genes. Our analysis identified various cell types, namely PCs, MCs, VCs, ECs, CCs, SMCs, and GCs, which are instrumental for studying the developmental dynamics of *Brassica* species’ shoot apex tissues (Fig. 1). We conducted a cross-species comparative analysis using published *Arabidopsis* vegetative shoot apex scRNA-seq data alongside our own findings, leading to the identification of highly correlated cell populations and conserved gene expressions between these two species (Fig. 3, Supplementary Data Fig. S5). Notably, the CC population exhibited a significant correlation across both species, with substantial parallels observed in the MC and SMC clusters. Furthermore, by integrating and comparing the scRNA-seq data from Chinese cabbage and *Arabidopsis*, we discovered numerous conserved genes involved in flowering and shoot development, particularly within the MC, SMC, and CC populations. This suggests a high degree of evolutionary conservation of these genes, reinforcing the value of cross-species studies in understanding plant development. For instance, the *STM* gene, a hallmark SMC marker in *Arabidopsis* [34], also plays a significant role in the SMCs of Chinese cabbage, validating our cell group categorization. Similarly, the *FLC* gene, known as a classical flowering suppressor in *Arabidopsis*, is involved in multiple flowering regulatory pathways [41]. *FLC* functions as a floral repressor gene, delaying the transition from vegetative to reproductive growth in plants. The role of *AtFLC* in *Arabidopsis* has been extensively characterized, particularly its primary targeting of three genes that influence flowering time: *FLOWERING LOCUS T* (*FT*), *SUPPRESSOR OF OVEREXPRESSION OF CONSTANS 1* (*SOC1*), and *FLOWERING LOCUS D* (*FD*) [3, 50]. Furthermore, at the level of epigenetic regulation, vernalization is controlled by H3K4me3 and H3K36me3 modifications, which form protein complexes that negatively regulate *AtFLC* in response to vernalization. Research on *AtFLC* has been progressively advancing. However, in Chinese cabbage, which possesses four *BrFLC* paralogs [42], a comprehensive annotation of *BrFLC* remains elusive [41]. Our cross-species analysis revealed that *BrFLC2* (BraA02g003340.3C) in the CC population of Chinese cabbage had a highly conserved expression pattern with the *FLC* gene in *Arabidopsis*. Functional validations of *BrFLC2* (Fig. 4) confirmed its role as a negative regulator of flowering in Chinese cabbage. Single-cell RNA sequencing provided a detailed perspective on *BrFLC2* expression in specific cell types within the shoot apex, highlighting its regulatory mechanisms. The conservation between *BrFLC2* and *FLC* underscores *BrFLC2*’s crucial role in vernalization and flowering regulation, presenting significant potential for breeding programs to develop cultivars with desired flowering traits.

We evaluated the pseudo-time trajectory of shoot apex development in Chinese cabbage (Fig. 5). In this process, DEGs among various cell populations were classified into five distinct states and bifurcated into two branches, reflecting the dynamic nature of the developmental trajectory. Intriguingly, our analyses of these states and branches revealed that a substantial majority of genes associated with flowering and shoot development were predominantly located in branch 1 and state 5, corresponding to the CC population (Supplementary Data Fig. S6E). This observation aligns with findings in *Arabidopsis*, where *FT*, a key component of florigen, has been shown to transmit photoperiodic flowering signals from leaf companion cells to the shoot apex [51]. This led us to hypothesize that the enrichment of flowering- and shoot development-related genes in the CC population of Chinese cabbage might similarly signal the translocation of specific flowering cues from companion cells to the shoot apex and promote bolting and flowering. To substantiate our hypothesis, we conducted additional pseudo-time trajectory analyses focusing on the MC, SMC, and CC groups. These analyses further supported our initial speculation. Additionally, examining the distribution of relevant flowering and shoot development genes (Fig. 6) allowed us to conclude confidently that our hypothesis was accurate. During the seed germination stage, most of the genes pertinent to Chinese cabbage are concentrated in the SMC population. Subsequently, as Chinese cabbage growth progresses, these genes likely transition to MCs and CCs, thereby facilitating rapid bolting and flowering. This comprehensive analysis not only elucidates the developmental dynamics within the shoot apex of Chinese cabbage but also underscores the critical role of cellular heterogeneity in the orchestration of key developmental processes.

Additionally, our comparative analysis shows the transformative impact of scRNA-seq in revealing the cellular mechanisms of vernalization in Chinese cabbage. This novel application of single-cell technology in the study of vernalization provides new opportunities for crop improvement and breeding strategies, especially in terms of manipulating flowering times and enhancing stress resistance. In our comparative analysis of Chinese cabbage shoot apexes under varying vernalization treatments, we observed that while vernalization did not significantly alter the intrinsic characteristics of cell types, it caused significant alterations in the relative proportions of cell-type-specific gene expression. Notably, the count of single cells in the vernalized samples was substantially lower than that in the non-vernalized samples (Supplementary Data Fig. S7). This discrepancy, alongside the observed consistency in cell types and frequencies, aligns with findings from previous studies [46, 47], suggesting that the reduced cell number in vernalized samples is likely attributable to low-temperature treatment. Thus, vernalization treatment adversely affects the status and quantity of single cells in the Chinese cabbage shoot apex. By conducting scRNA-seq on samples subjected to different treatments, we found heterogeneity and consistency in gene expression patterns post-vernalization. A greater number of DEGs were identified in the PC and MC populations compared with the others, with a notably lower number of DEGs in the GC population (Fig. 7B). Furthermore, the expression patterns of the five key genes involved in the vernalization pathway varied across different cell types in the two sample sets (Fig. 7C). Intriguingly, the CC population was enriched with DEGs related to the *VRN1*, *FLC*, and *FT* genes. This finding corroborates our previous observation that the CC population is a hub for genes involved in flowering, highlighting its critical role in the flowering pathway of Chinese cabbage. Previous studies have indicated that vernalization does not affect the expression of *VRN1* and *VRN2*, but under vernalizing conditions *VRN1* and *VRN2* can repress the expression of *FLC* genes [4, 48]. However, our findings suggest that the expression patterns of *VRN1* and *VRN2* varied across different cell populations in the NV and V25 samples. This indicates that while vernalization may not significantly alter the overall expression of *VRN1* and *VRN2* genes in the entire sample, it might affect the expression of *VRN1* and *VRN2* in specific cell types. Additionally, among the 60 DEGs identified across various cell populations, excluding GC, five TFs were prominent (Fig. 7E). Cell differentiation is often governed by transcriptional regulation, and the expression of these five TFs varied among samples and cell populations under different vernalization treatments, providing valuable insights for future vernalization research. This observation highlights the importance of transcriptional regulation in determining cell differentiation and modulating the developmental trajectory of individual cell types [14]. This analysis shows that vernalization differentially affects cell types within the shoot apex, thus advancing our understanding of the cellular mechanisms governing flowering genes in Chinese cabbage. Our findings not only provide a deeper understanding of the molecular mechanisms of vernalization in Chinese cabbage but also pave the way for future research focused on optimizing crop development and flowering through advanced single-cell technologies. This study serves as a stepping stone for further exploration into the functional interactions of key TFs and shoot apex development under diverse environmental conditions. The exploration and GO annotation of DEGs related to shoot development and flowering revealed distinct enrichment patterns in various cell types. Among these findings, the identification of the flowering-suppressor gene, *SOBIR1* (Fig. 7F), opens up a new avenue for research on Chinese cabbage and its flowering traits. These insights are invaluable for future agricultural practices and breeding programs, as they provide a deeper understanding of the genetic and cellular foundations of plant development and vernalization.

## Conclusion

In summary, our study successfully established a novel and comprehensive gene expression profile of the Chinese cabbage shoot apex at a single-cell resolution. This achievement not only enhances precision in cell type identification and characterization using various cell markers but also lays a foundational framework for future research in cellular biology and genomics. Furthermore, our comparative analysis of the shoot apex in both Chinese cabbage and *Arabidopsis* has shed light on conserved and divergent aspects of cell type function and development in these species. This comparison offers a fresh perspective on the role and evolutionary dynamics of cell types in Chinese cabbage. One of the pivotal discoveries of our research is the proposal that the CC population in plants, particularly in Chinese cabbage, may be a crucial reservoir of a large number of genes involved in flowering. This finding highlights the importance of the CC population and suggests that it warrants more in-depth exploration in future studies. Additionally, the pseudo-time trajectory and DEG analyses of samples treated under different conditions have provided invaluable resources. These insights are pivotal for elucidating the function and evolutionary pathways of vernalization-related genes at the single-cell level. Moreover, our findings have significant implications for the breeding of Chinese cabbage. By understanding the intricacies of gene expression associated with key developmental stages and processes, breeders can develop more efficient strategies for cultivating Chinese cabbage varieties with desired traits. This research not only contributes to the fundamental understanding of plant developmental biology but also offers practical applications in agricultural biotechnology, potentially leading to the enhancement of crop quality and yield.

## Materials and methods

### Plant materials and growth conditions

Germination and plumule-vernalization treatments were applied to the bolting-resistant Chinese cabbage DH line ‘Ju Hongxin’ (JHX) [52]. Eight hundred uniformly healthy JHX seeds were carefully selected, sterilized with water, and arranged in Petri dishes lined with two layers of filter paper. Each dish contained 40 seeds, and there were a total of 20 dishes, equally divided for vernalization and non-vernalization treatments. To expedite germination, the seeds were incubated in a climate chamber set at a constant temperature of 25°C with 16 h of light per day for 2 days. Once the radicles emerged, the dishes were split into two groups: one group was moved to a vernalization chamber at 4°C with a 22/2-h light/dark cycle and 150 μmol m^−2^ s^−1^ light intensity for 25 days (V25 treatment), and the other group was kept in an artificial climate chamber at 25°C with the same light/dark cycle and light intensity for 25 days (N25 treatment) (Fig. 1A).

### Preparation of shoot apex meristem samples for scRNA-seq

The Chinese cabbage vegetative shoot apex, consisting of the SAM and leaf primordium, posed challenges in isolating the SAM. Utilizing precision cutting and microscopic techniques, we collected ~200 shoot apices from both N25 and V25 treatments to enrich the SAM content. The shoot apex tissue was digested in an RNase-free enzyme solution containing 1.5% cellulase R10, 0.75% macerozyme, 0.1% pectinase, 1% cellulose RS, 0.5 M mannitol, 10 mM MES (pH 5.7), 0.5% PVP-40, 2 mM KCl, 10 mM CaCl_2_, and 0.5% BSA at room temperature for 2 h. The cells were subsequently filtered through a 40-μm mesh and their viability was assessed using trypan blue staining. The cell density was determined using a hemocytometer and a light microscope (DM2000, Leica, Germany). Finally, the protoplasts were suspended in a 0.4 M mannitol solution, and the concentration was adjusted to 1500–2000 cells/μl, resulting in the formation of shoot apex cell suspensions.

### scRNA-seq library construction and data processing

Chinese cabbage shoot apex cell suspensions were processed using the Chromium Single Cell Instrument (10× Genomics) and libraries were generated and sequenced using Chromium Next GEM Single Cell 3′ Reagent Kits v3.1. The resulting Illumina-ready sequencing libraries followed the Single Cell 3′ Protocol [53].

Raw BCL files were converted to FASTQ format, followed by alignment and quantification with 10× Genomics Cell Ranger software (version 3.1.0). Reads with low-quality barcodes and unique molecular identifiers (UMIs) were removed and then mapped to the Chinese cabbage genome (version 3.0, http://brassicadb.cn). Only unique reads with at least 50% exon overlap were counted as UMIs. UMI sequences underwent error correction, and valid barcodes were identified using the EmptyDrops method [54] before quantification. Cell-by-gene matrices were created through UMI counting and barcode retrieval, and each sample’s matrix was separately loaded into Seurat (version 3.1.1) [55] for clustering, quality control, and scRNA-seq data analysis. Cells exhibiting unusually high UMIs (≥8000) or mitochondrial gene percentages (≥10%) were excluded. To correct for batch effects and behavioral conditions, the Harmony algorithm [56] was applied to achieve a batch-corrected result using PCA embedding of cells and their batch assignments. The expression matrix was scaled, and dimensionality was reduced with principal component analysis (PCA). Methods such as *t*-distributed stochastic neighbor embedding (*t*-SNE) [57, 58] and uniform manifold approximation and projection (UMAP) [59] were used to group cells with similar local neighborhoods in both high- and low-dimensional spaces. Log-normalized matrices were used in the SingleR R package to annotate cell types by correlating reference gene expression with single-cell data [60].

Gene expression in clusters was compared with other cells using the Wilcoxon rank sum test [61]. Mean expression in cell subpopulations was quantified using TPM (transcripts per kilobase of exon model per million mapped reads). TPM values were calculated as follows: TPM_A_ = UMI_A_ ÷ UMI_Total_ × 10 000, where TPM_A_ represents the TPM value of gene A in the target cell, UMI_A_ is the number of UMIs of gene A, and UMI_Total_ is the total number of UMIs in the target cell. GO enrichment analysis identified significantly enriched GO terms of DEGs relative to the genomic background, filtering DEGs based on biological functions [62]. KEGG analysis pinpointed significantly enriched metabolic or signaling pathways in DEGs compared with the genome-wide background [63]. Differential analysis among groups was conducted based on cell subgroups and sample data, using Seurat software version 3.1.1 [55]. Parameters were set with |log_2_FC| ≥ 0.36 and a minimum cell proportion expressing the target gene in any group ≥0.1. Significance was assessed using the MAST hurdle model [64], and the Benjamini–Hochberg method in Seurat [55] was applied for multiple testing correction. Genes with an adjusted *P*-value ≤0.05 were considered significantly differentially expressed.

### Pseudo-time trajectory analysis

Monocle 2 software [45] can use the signals of gene expression levels in all cells, based on the pseudo-time values of each cell, to screen for DEGs along the timeline, looking for critical genes related to the process of development and differentiation. Based on Monocle 2, the cells were arranged on a cell trajectory according to the pseudo-time change, and the cell differentiation relationship during development was simulated [65]. Monocle 2 models the locus as a tree with a ‘root’ at one end and a ‘leaf’ at the other. A cell moves from the root up the trunk to the first branch, choosing a path and continuing until it reaches a leaf. Differential expression analysis using Monocle 2 identified DEGs between the same cell groups in N25 and V25 and assessed the statistical significance of these findings.

### Identification of one-to-one orthologs

Protein sequences of Chinese cabbage and *Arabidopsis* were downloaded from BRAD and TAIR to identify orthologous genes. Using the OrthoMCL algorithm [36], similar sequences were clustered with an all-against-all BLASTP strategy (e-value 1^−e5^). The clusters were divided into one-to-one, one-to-many, and many-to-many groups. Only one-to-one homolog pairs were used (Supplementary Data Table S7), as one-to-many and many-to-many groups are not functionally equivalent.

### Interspecies scRNA-seq data comparison

Two methods were used for cross-species analysis. The first involved calculating pairwise correlations between cell types using the gene specificity index equation (Fig. 3C), which has been applied to various animal and plant species [66]. Seurat’s average expression function and gene specificity index equation were applied following the methods outlined by Zhang *et al*. [11]. The second approach combined scRNA-seq data from Chinese cabbage and *Arabidopsis* shoot apexes. For this purpose, we first selected the published *Arabidopsis* shoot apex data shoot4 (BioProject PRJCA003094) to construct an *Arabidopsis* shoot apex cell atlas. By selecting the reference genome (Ensembl release 54) and using Cell Ranger for fastq for data processing, 23 828 cells and 31 136 genes were obtained. Cell clustering of the *Arabidopsis* shoot apex using similar parameters as Seurat was performed, as described above, to yield cell clusters (Fig. 3A). The scRNA-seq datasets of Chinese cabbage and *Arabidopsis* shoot apexes were later integrated by canonical correlation analysis (CCA) in Seurat (Supplementary Data Fig. S5A). Twenty cell clusters were clustered after eliminating cross-species batch effects (Supplementary Data Fig. S5B and S5C).

### RNA fluorescence *in situ* hybridization method

cDNA from the N25 stage was used to clone the full-length cDNAs of *PNSL1* (BraA05g006520.3C) and *SUC2* (BraA07g014520.3C). Custom-labeled nucleic acid probes (Supplementary Data Table S21) were designed to detect the cellular localization of PNSL1 and SUC2. Chinese cabbage shoot apex sectioning and fluorescence *in situ* hybridization (FISH) methods followed those described by Dai *et al*. [67].

### Experimental processes for the *BrFLC2* functional study

*BrFLC2* was cloned from a Chinese cabbage shoot apex cDNA library. FLC protein sequences were retrieved from the NCBI database for various species. These sequences were then used to construct a phylogenetic tree with MEGA V7.0 [68]. The T-DNA insertion mutant *flc* (SALK_072590) was sourced from the *Arabidopsis* Biological Resource Centre (ABRC), and homozygous *flc* mutants were confirmed by PCR for overexpression studies. The *BrFLC2* coding sequence, excluding the termination codon, was cloned and fused with GFP in the 2300-eGFP vector to create the BrFLC2-eGFP plasmid. The 35S:*BrFLC2*-eGFP construct and p2300-35S-H2B-mCherry-OCS (a plasmid that marks the position of the cell nucleus) were separately transformed into GV3101. The infiltration solution containing 35S::*BrFLC2* was introduced into the leaves of 30-day-old *Nicotiana benthamiana* (tobacco) seedlings. After 48–60 h, fluorescence signals were examined using a confocal laser scanning microscope (LSM780, Zeiss, Germany). The *BrFLC2* coding sequence was cloned into the pBI121 vector to create the 35S::*BrFLC2* plasmid, which was then transfected into GV3101 and introduced into *Arabidopsis* plants using the floral dip method [69]. The screening of overexpression lines from T0 to T3 followed the method described by Dai *et al*. [67].

After 10 days on 1/2 MS medium, OX (overexpression), *flc Arabidopsis* and WT (Columbia wild type) seeds were transferred to potting soil and grown in a controlled greenhouse with a 10/14-h light/dark cycle and 150 μmol m^−2^ s^−1^ light intensity. This soil growth phase was considered day 0. Key stages were recorded: bolting was defined when the stem reached 2 cm, flowering was marked by the first flower fully blooming, and seed-setting was noted upon the formation of the first pod. Each treatment was replicated 10 times to ensure data reliability and reproducibility. The OX, *flc*, and WT lines were maintained under standard growth conditions. After 30 days, shoot apex tissues from *Arabidopsis* were collected, flash-frozen in liquid nitrogen, and stored at −80°C. RNA was extracted from the samples and reverse-transcribed into cDNA. Quantitative RT–PCR was then performed to determine the expression levels of *BrFLC2*, *AtFT*, and *AtSOC* in each line. The *BrFLC2* coding sequence was cloned into the pGBKT7 vector, and a bait self-activation test confirmed that *BrFLC2* did not exhibit self-activation. The Chinese cabbage library was then cloned into the pGADT7 vector as prey. Y2H library screening was conducted following the manufacturer’s protocol (YH2012, Coolaber, China). To verify the interactions, *BrFLC2*, *BrTIL*, and *BrMSI4* coding sequences were cloned into pGBKT7 and pGADT7 vectors. These sequences were then fused with the binding domain (BD) and activation domain (AD) to form bait and prey constructs. The constructs were introduced into the yeast strain Y2H-Gold and incubated on DDO/X medium (SD/−Leu−Trp) with X-α-Gal at 30°C for 3 days. Positively transformed clones were used to inoculate TDO/X medium (SD/−Trp/−His/−Leu) containing X-α-Gal to test protein interactions. The luciferase LUC assay constructors, nluc-Br*FLC2* and cluc-*BrMSI4*, were transferred into GV3101 and transiently transformed into tobacco leaf epidermal cells. After 36–48 h, fluorescein spray (100 μM; Promega) was applied to the abaxial surface of the leaves, which were kept under dark conditions for 5 min, and then fluorescence was observed. The In Vivo Plant Imaging System (Berthold Technologies, Germany) was used to capture images. All primers are listed in Supplementary Data Table S21.

## Supplementary Material

Web_Material_uhae214

## Data Availability

The raw sequence data from this research have been submitted to the Genome Sequence Archive (GSA: CRA014199). The GSA is maintained by the National Genomics Data Center, which is part of the China National Center for Bioinformation and the Beijing Institute of Genomics. These institutions are affiliated with the Chinese Academy of Sciences. The data are publicly accessible at the following URL: https://ngdc.cncb.ac.cn/gsa.

## Supplementary information

The supplementary tables associated with this study have been uploaded to Zenodo for open access. These tables include detailed statistical data and analysis results that support the findings of this paper. The data can be accessed via the following DOI: https://doi.org/10.5281/zenodo.12759721. Supplementary figures are available at *Horticulture Research* online.
