# Supplementary material for: Single-cell transcriptomic analysis of flowering regulation and vernalization in Chinese cabbage shoot apex
Source: Hortic Res. 2024 Jul 30;11(10):uhae214. doi: 10.1093/hr/uhae214 (PMC11464683; doi:10.1093/hr/uhae214)
Supplement: Web_Material_uhae214 [file web_material_uhae214.zip › Supplemental Figures.docx]

**Supplemental Fig. S1**

**
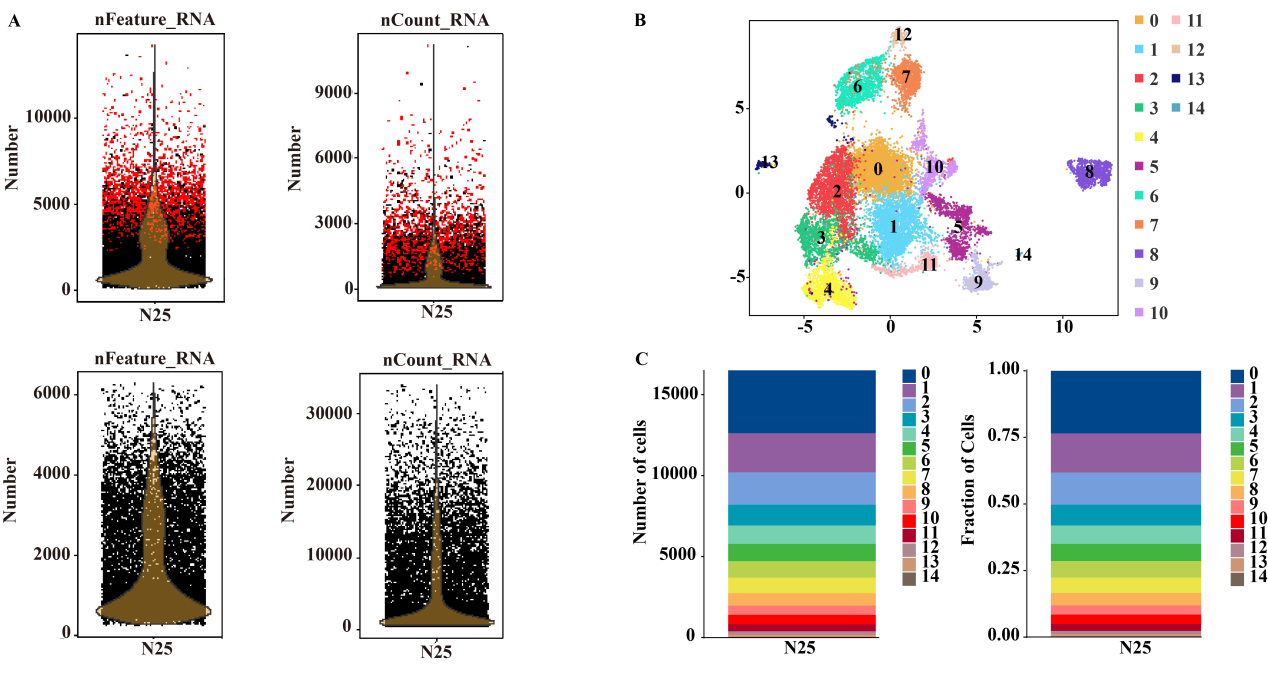
**

**Supplemental Fig. S2**

**
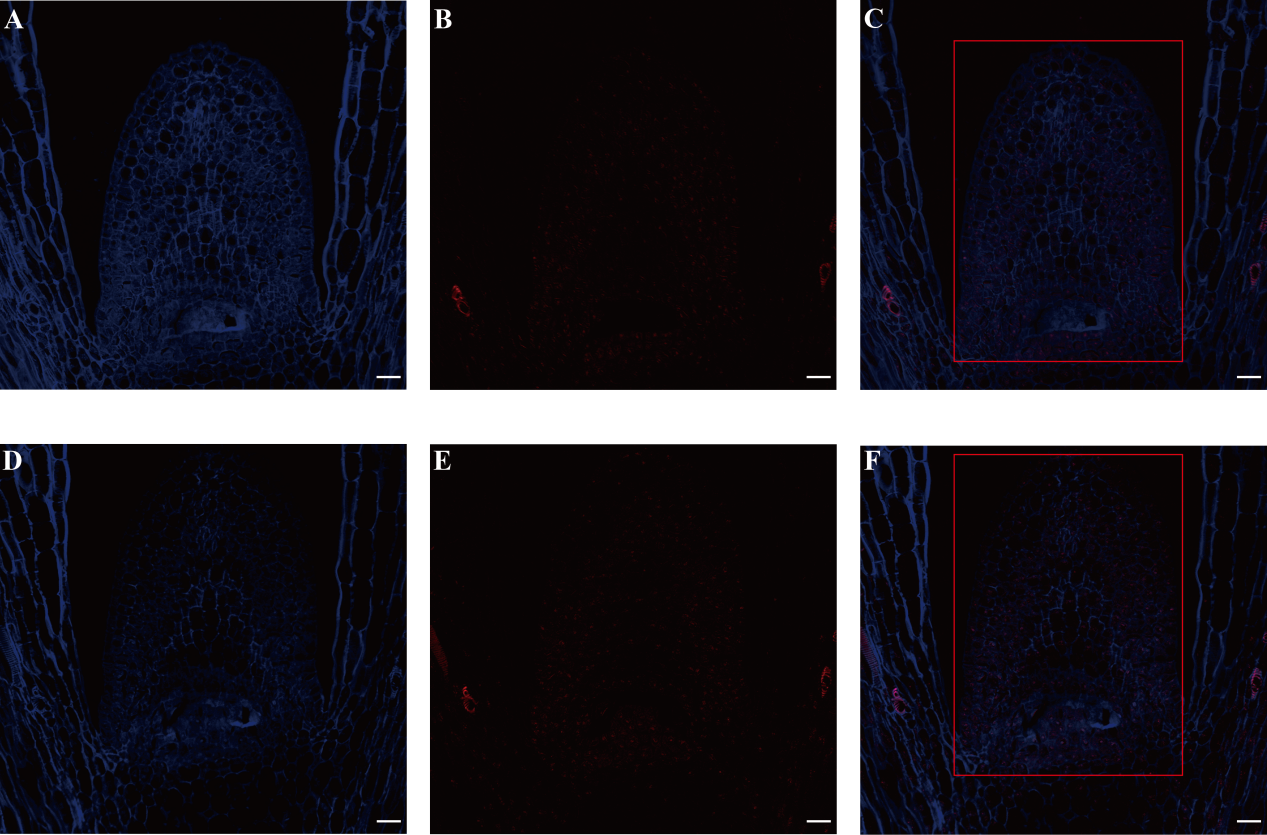
**

**Supplemental Fig. S3**

**
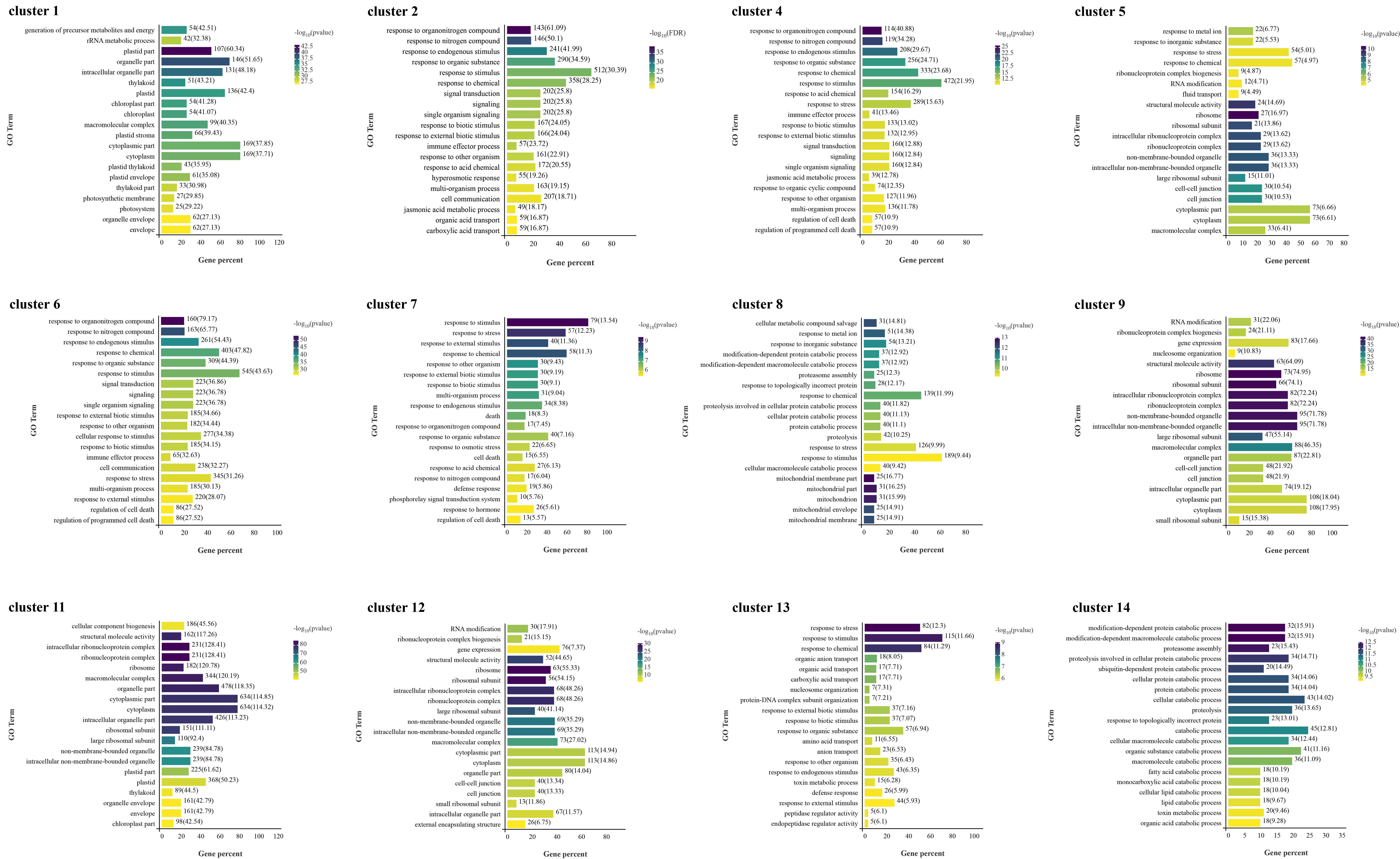
**

**Supplemental Fig. S4**

**
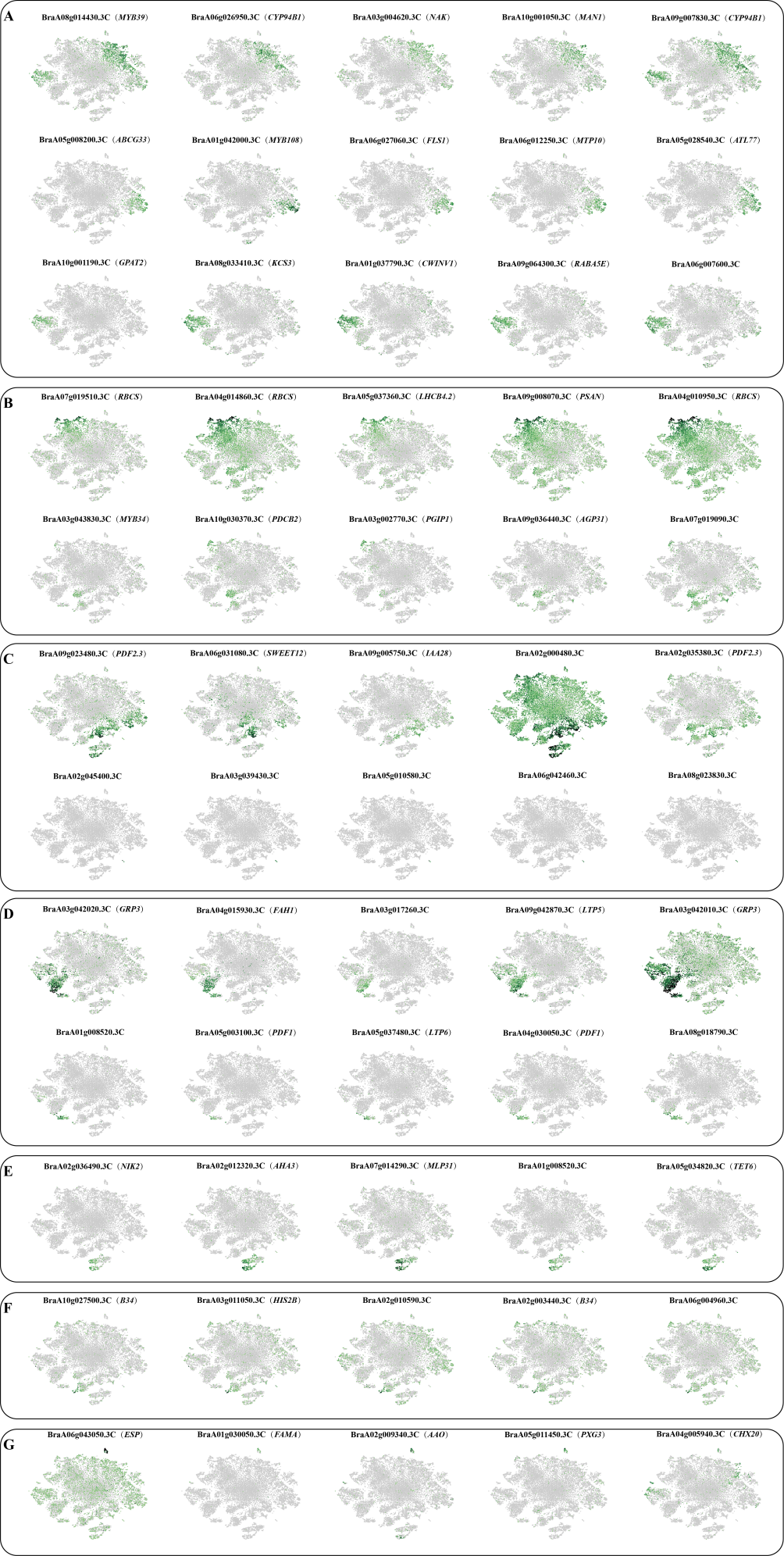
**

**Supplemental Fig. S5**

**
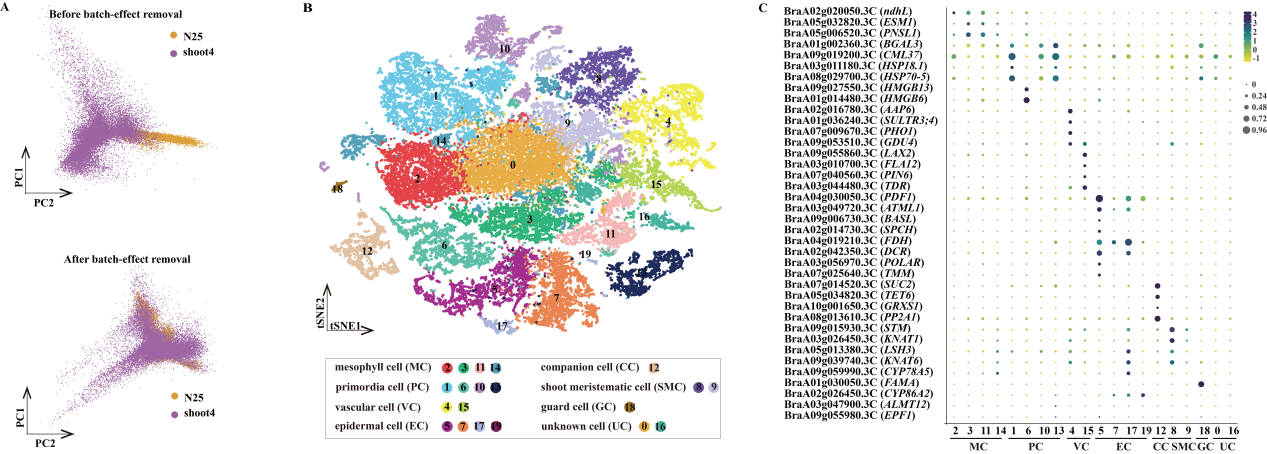
**

**Supplemental Fig. S6**

**
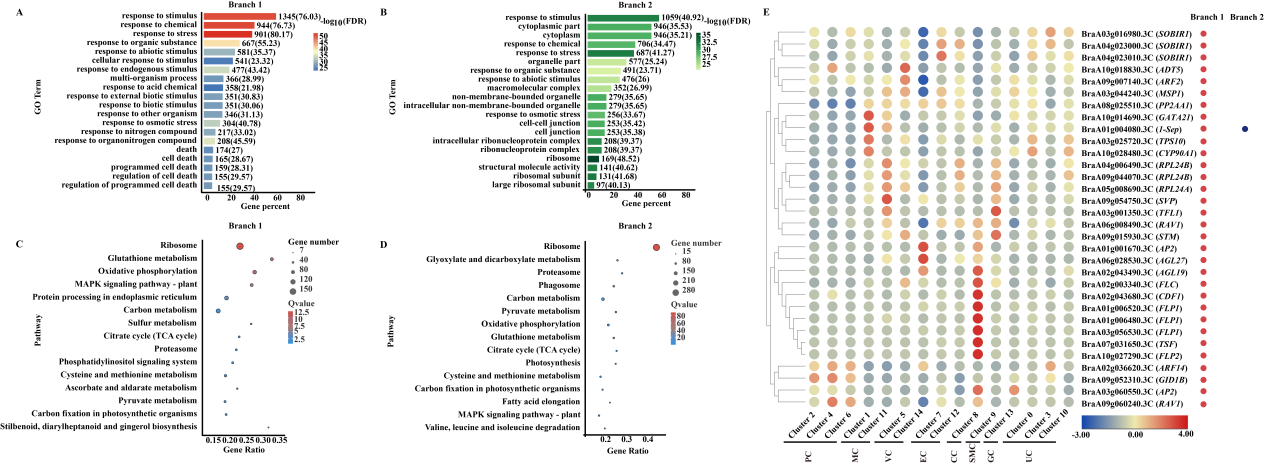
**

**Supplemental Fig. S7**

**
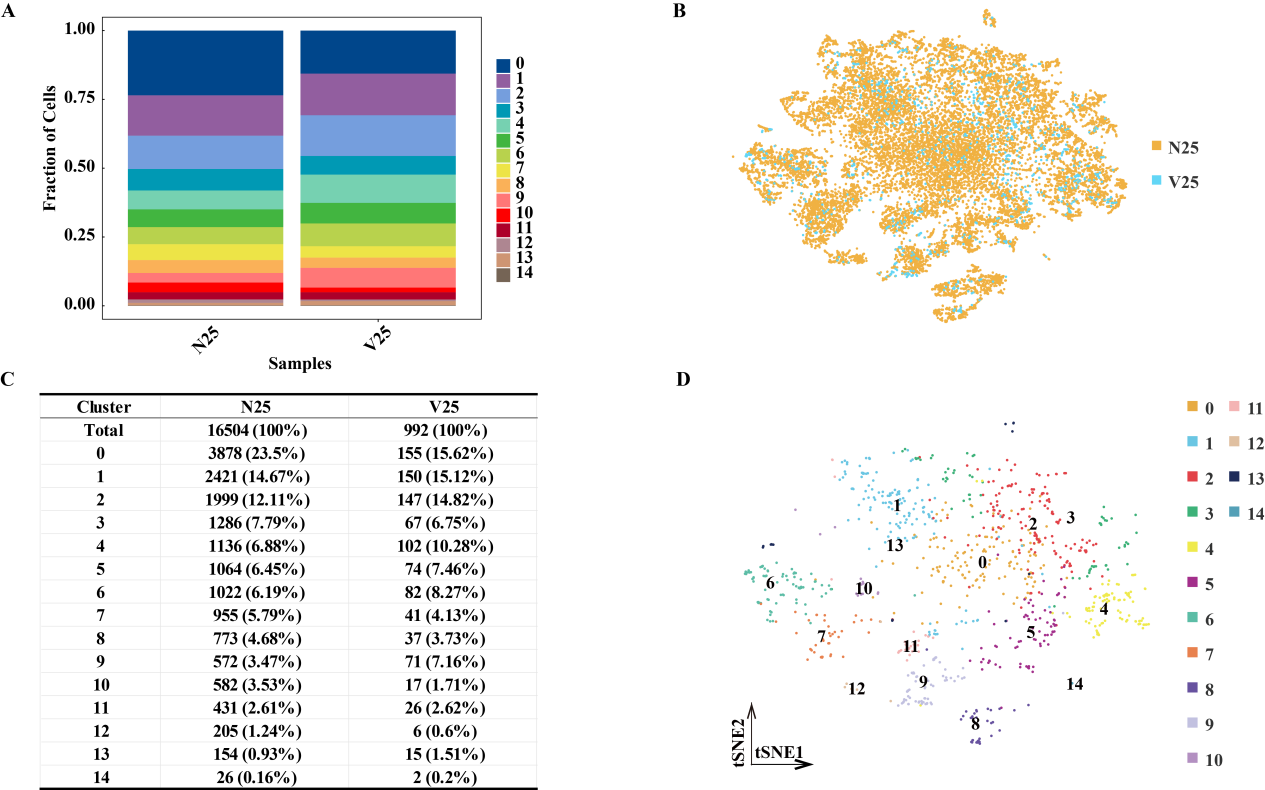
**

**Supplemental Fig. S8**

**
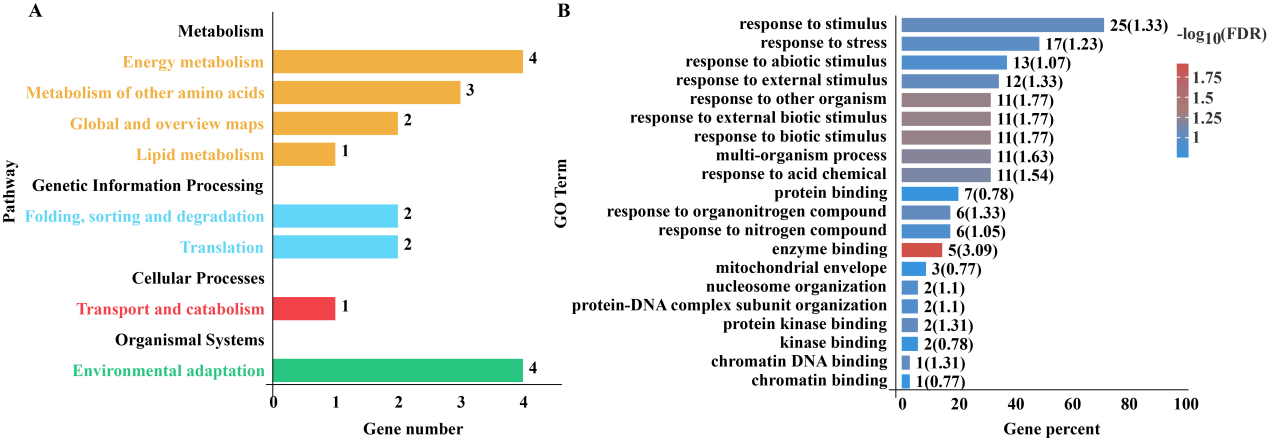
**
